# COVID-19 patients and Dementia: Frontal cortex transcriptomic data

**DOI:** 10.1016/j.dib.2021.107432

**Published:** 2021-09-29

**Authors:** Maria Garofalo, Stella Gagliardi, Susanna Zucca, Cecilia Pandini, Francesca Dragoni, Daisy Sproviero, Orietta Pansarasa, Tino Emanuele Poloni, Valentina Medici, Annalisa Davin, Silvia Damiana Visonà, Matteo Moretti, Antonio Guaita, Mauro Ceroni, Livio Tronconi, Cristina Cereda

**Affiliations:** a Genomic and post-Genomic Unit, IRCCS Mondino Foundation, Via Mondino, 2, Pavia 27100, Italy; b Department of Biology and Biotechnology "L. Spallanzani", University of Pavia, Pavia, Italy; c EnGenome SRL, Pavia 27100, Italy; d Department of Neurology and Neuropathology, Golgi-Cenci Foundation & ASP Golgi-Redaelli Abbiategrasso, Milano, Italy; e Department of Public Health, Experimental and Forensic Medicine, Unit of Legal Medicine and Forensic Sciences “A. Fornari”, University of Pavia, Pavia, Italy; f Department of Brain and Behavioral Sciences, University of Pavia, Pavia, Italy; g Department of General Neurology, IRCCS Mondino Foundation, Pavia, Italy; h U.O. Medicina Legale, IRCCS Mondino Foundation, Pavia, Italy

**Keywords:** SARS-CoV-2, Transcriptomics, Gene expression, Brain

## Abstract

Since the association of SARS-Cov-2 infection with Nervous System (NS) manifestations, we performed RNA-sequencing analysis in Frontal Cortex of COVID-19 positive or negative individuals and affected or not by Dementia individuals. We examined gene expression differences in individuals with COVID-19 and Dementia compared to Dementia only patients by collecting transcript counts in each sample and performing Differential Expression analysis. We found eleven genes satisfying our significance criteria, all of them being protein coding genes.

These data are suitable for integration with supplemental samples and for analysis according to different individuals’ classification. Also, differential expression evaluation may be implemented with other scientific purposes, such as research of unannotated genes, mRNA splicing and genes isoforms.

The analysis of Differential Expressed genes in COVID-19 positive patients compared to non-COVID-19 patients is published in: S. Gagliardi, E.T. Poloni, C. Pandini, M. Garofalo, F. Dragoni, V. Medici, A. Davin, S.D. Visonà, M. Moretti, D. Sproviero, O. Pansarasa, A. Guaita, M. Ceroni, L. Tronconi, C. Cereda, Detection of SARS-CoV-2 genome and whole transcriptome sequencing in frontal cortex of COVID-19 patients., Brain. Behav. Immun. (2021). https://doi.org/10.1016/j.bbi.2021.05.012.

## Specifications Table

**Table:** 

Subject	Omics: Transcriptomics
Specific subject area	Bulk Whole RNA-sequencing and Differentially Expressed Genes analysis
Type of data	Table Graph Figure
How data were acquired	-Illumina NextSeq 500 Sequencer, -llumina bcl2fastq2 (Version 2.17.1.14 - http://support.illumina.com/downloads/bcl-2fastq-conversion-software-v217.html) -STAR/RSEM (1.3.3) -R (v4.0.2) packages: DESeq2 (1.30.0) enrichR (2.1)
Data format	Raw Analyzed Filtered
Parameters for data collection	We considered four conditions for data collection: (1) COVID-19 individuals with Dementia; (2) NO COVID-19 individuals with Dementia; (3) COVID-19 individuals without Dementia; (4) NO COVID-19 individuals without Dementia.
Description of data collection	Data were collected through bulk RNA-sequencing of total RNA extracted from post-mortem Frontal Cortexes.
Data source location	(1) Institution: IRCCS Mondino Foundation City: Pavia Country: Italy
Data accessibility	Repository name: GEO (Gene Expression Omnibus)-NCBI Data identification number: GSE164332 Direct URL to data: https://www.ncbi.nlm.nih.gov/geo/query/acc.cgi?acc=GSE164332
Related research article	S. Gagliardi, E.T. Poloni, C. Pandini, M. Garofalo, F. Dragoni, V. Medici, A. Davin, S.D. Visonà, M. Moretti, D. Sproviero, O. Pansarasa, A. Guaita, M. Ceroni, L. Tronconi, C. Cereda, Detection of SARS-CoV-2 genome and whole transcriptome sequencing in frontal cortex of COVID-19 patients., Brain. Behav. Immun. (2021). https://doi.org/10.1016/j.bbi.2021.05.012.

## Value of the Data

• We exploited Next Generation Sequencing technique for providing transcriptomic profiles in Frontal Cortex of both COVID-19 positive or negative individuals and affected or not by Dementia individuals. These screenings are important for the study of impact of current infectious disease on Central Nervous System, so-called NeuroCOVID-19, and on diverse elderly comorbidities, such as Dementia. The aim was to collect information concerning RNA alterations in the prefrontal cortex given its contribution in hemodynamic responses.
• These data can help in the study of molecular features of SARS-CoV-2 in the brain. Moreover, the dysregulation of specific pathways can be extrapolated from transcriptomic data making them a source of biomarkers.
• Versatility of both raw and analysed RNA-sequencing data lies in their suitability for several purposes, such as gene expression analysis, unannotated genes discovery, mRNA splicing investigation and genes isoforms study. *In addition, data in standard format, such as FastQ and BAM files, but also gene expression tables reporting raw counts, FPKM and TPM values, can be easily re-used and integrated with additional samples or exploited to refine the analysis with different individual classification.”*

## 1 Data Description

A summary of anagraphic and clinical feature of cases included in transcriptomic investigation is reported in Table 1. Individuals with Dementia were six, individuals with Dementia and COVID-19 were seven, two individuals had neither Dementia nor COVID-19 and two individuals had COVID-19 but not Dementia.

**Table 1:** Summary of the anagraphic and clinical characteristics of COVID-19 and NON-COVID-19 cases. Under COVID column, “+” stands for positive, “−” stands for negative.

CASE	COVID	AGE	SEX	PMD (hours)	DEMENTIA
COV1	+	74	F	168	Dem
COV3	+	87	M	168	Dem
COV4	+	67	M	120	No Dem
COV5	+	94	F	72	Dem
COV7	+	80	F	360	No Dem
COV8	+	83	F	312	Dem
COV9	+	92	M	144	Dem
COV10	+	81	M	168	Dem
COV6	+	90	F	264	No Dem
BB247	−	104	F	6	Dem
BB236	−	80	M	15	Dem
BB109	−	79	M	16	No Dem
BB47	−	78	F	8	Dem
BB271	−	84	F	2	Dem
BB138	−	85	F	15	Dem
BB120	−	84	M	10	Dem
BB118	−	79	M	3	No Dem

*Abbreviations:* PMD = Post Mortem Delay; Dem = Dementia; No Dem = No Dementia.

In Supplementary Table 1, the counts of each gene (specified as Ensembl ID) are indicated for each sample submitted to sequencing.

**Fig. 1:**
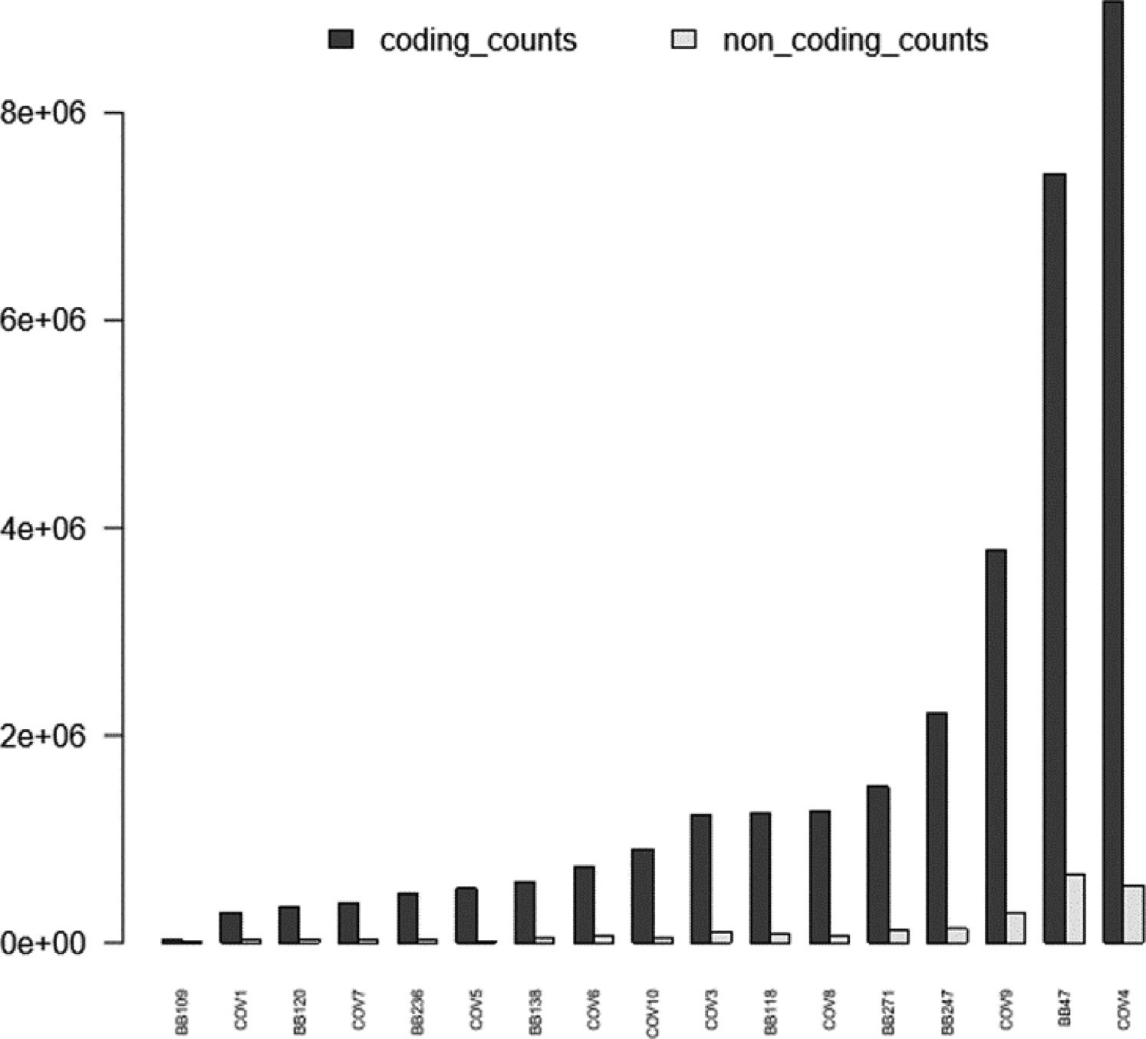
Coding and non-coding RNA counts after deep sequencing, demultiplexing and alignment.

The amount of both coding and non-coding counts was evaluated for each sample and as visible in Fig. 1, coding ones were the most abundant. This result is in accordance with the currently available knowledge about non-coding transcripts that result to be globally less expressed than coding ones within the cell [1,2]. BB109 was nonuniform in terms of counts abundancy and did not pass quality check, thus this sample was excluded from further analysis.

**Fig. 2:**
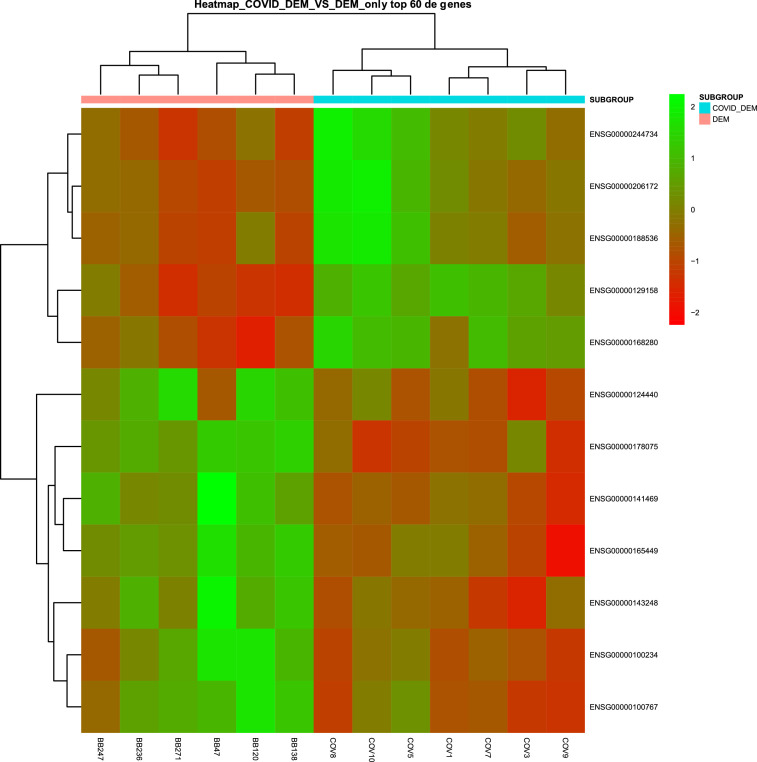
Heatmap of top 11 Differentially Expressed (DE) genes. Samples from COVID-19 positive individuals with Dementia (*n* = 7) are marked in light blue, while patients with Dementia (*n* = 6) are marked in pink. (For interpretation of the references to color in this figure legend, the reader is referred to the web version of this article).

A differential expression analysis of genes was performed. We compared the group of individuals with COVID-19 and Dementia (*n* = 7) versus those with Dementia only (*n* = 6). In order to evaluate the clustering resulting from this analysis, we represented in the Heatmap in Fig. 2 all the deregulated genes. The list of genes considered significant in this analysis is available in Supplementary Table 2. We found dysregulated 11 genes, 4 up-regulated and 7 down-regulated. All of them were protein coding. In this table Ensembl ID, base mean, log2FoldChange, lfcSE, stat, *P*-value, adjusted *P*-value, gene name, gene biotype and gene source are indicated.

We also performed differential expression analysis of genes considering COVID-19 patients without Dementia (*n* = 2) versus COVID-19 negative individuals without Dementia (*n* = 1), but we found no significantly deregulated genes observing our filtering criteria as reported in Supplementary Table 2.

**Fig. 3:**
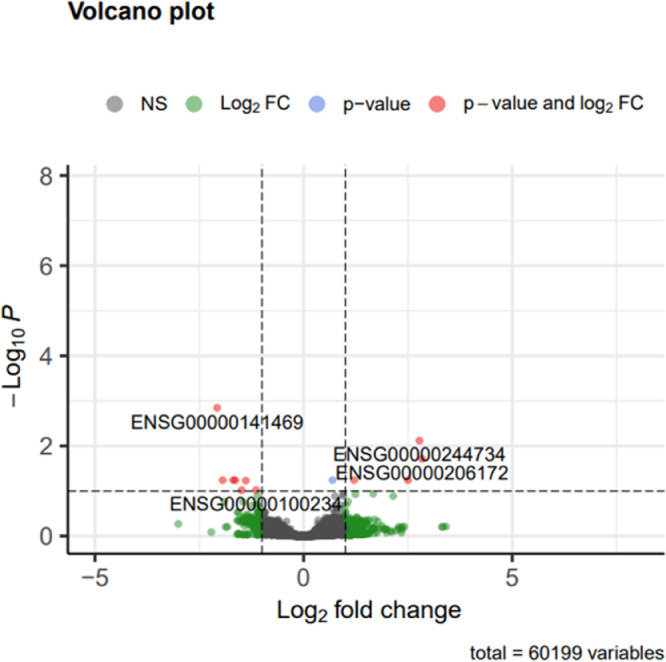
Volcano plots obtained from DE analysis of patients with Dementia who died with COVID-19 (*n* = 7) versus patients with Dementia only (*n* = 6). The most upregulated genes are towards the right, the most downregulated genes are towards the left, and the most statistically significant genes are towards the top. Red dots represent significant up- and down-regulated genes which have |log2(fold change)|≥ 1 and a *p*-value ≤ 0.1. Blue, green and grey dots represent non-significant DE detected genes, because they do not satisfy both requirements. The top 4 DE genes are labelled (Ensembl ID). (For interpretation of the references to color in this figure legend, the reader is referred to the web version of this article).

The volcano plot in Fig. 3 shows statistical significance (*P*-value) versus magnitude of change (fold change) of differential expressed (DE) genes in COVID-19 and Dementia individuals (*n* = 7) versus individuals with Dementia only (*n* = 6). The number of genes with |log2(fold change)|n 1 that are also statistically significant is low.

## 2 Experimental Design, Materials and Methods

Autoptic human brain samples were used for collecting these data. RNA was isolated by Trizol reagent (Life Science Technologies, Italy) according to the manufacturer's instructions and processed as described in Gagliardi et al. [1].

Starting from 1 µg of total RNA, sequencing libraries were prepared with the CORALL Total RNA-Seq Library Prep Kit (Lexogen, Vienna, Austria) and sequenced on an Illumina NextSeq 500 Sequencing (Illumina, San Diego, CA) as described in Gagliardi et al. [1] . FastQ files were generated via llumina bcl2fastq2 (Version 2.17.1.14 - http://support.illumina.com/downloads/bcl-2fastq-conversion-software-v217.html) starting from raw sequencing reads produced by Illumina NextSeq sequencer.

**Fig. 4:**
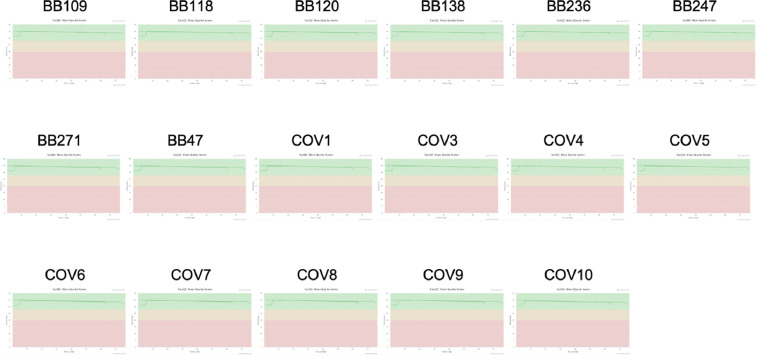
Quality assessment of FASTQ sequences data for paired end and right reads. Each plot shows the mean quality value across each base position in the read for all the analyzed samples.

**Fig. 5:**
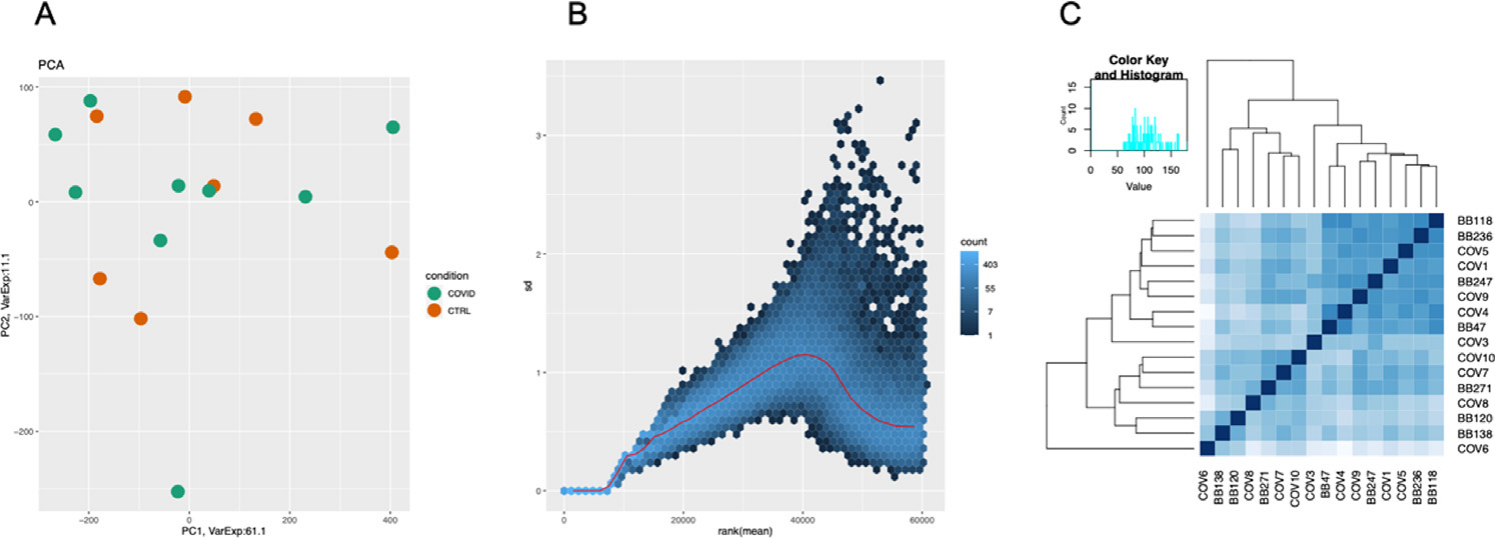
Panel A shows Principal component analysis result on the whole dataset. Panel B shows an estimate of the dispersion parameter for each gene. In Panel C, the heatmap of the sample-to-sample distance is shown. It was obtained with DeSeq2 package on regularized-logarithm transformed counts. Color code is reported above the heatmap.

Quality of individual sequences were evaluated using MultiQC software (https://multiqc.info/) after adapter trimming with cutadapt software. UMI sequences were marked and deduplicated with UMI-tools software [2] [UMItools]. Per base sequence quality plots, showing the mean quality value across each base position in the read are shown in Fig. 4. Gene and transcript intensities and differential expression analysis for mRNA and non coding RNAs were computed as in Gagliardi et al. [1]. Human genome reference used for the alignment was GRCh38 (Gencode release 36), containing the up-to-date records for both coding and non coding RNAs. Coding and non coding genes were considered differentially expressed and retained for further analysis with |log2(disease sample/healthy control)| ≥ 1 and a FDR ≤ 0.1. We imposed minimum |Log2FC| of 1 and a FDR lower than 0.1 as thresholds to differentially expressed genes. Inter- and intra-group variability was assessed and shown in Fig. 5. On average, 29.2 M reads were available for each sample and 22.7 M reads were aligned against the reference genome (average overall alignment rate: 77.9%). Input reads number, average read length, number of aligned reads and alignment rate are reported in Table 2 for each sample. Transcripts with a count value of at least 5 were retained for differential expression analysis. On average, 16734.8 coding genes and 5370.6 non coding genes resulted to be expressed in each sample.

**Table 2:** For each sample indicated in “Sample_name” column, the total number of input reads, the average read length, the number of reads uniquely mapped to the reference genome and the overall alignment rate are reported.

Sample_name	Number of input reads	Average input read length	Uniquely mapped reads num	Uniquely mapped reads percentage
BB109	2043229	136	1465303	71.72%
BB118	7818855	136	6856173	87.69%
BB120	41915410	130	34790052	83.00%
BB138	14439548	136	12677228	87.80%
BB236	8773432	137	7747656	88.31%
BB247	72990663	133	62361438	85.44%
BB271	52689242	131	43149563	81.89%
BB47	42194239	135	36628979	86.81%
COV1	3558188	136	2844285	79.94%
COV10	37740062	132	28378973	75.20%
COV3	37337535	126	13450528	36.02%
COV4	35786418	134	29279441	81.82%
COV5	2309778	136	1981240	85.78%
COV6	39798363	134	33643489	84.53%
COV7	11548099	136	9483185	82.12%
COV8	38311594	131	24019227	62.69%
COV9	46815183	132	37817645	80.78%

## Ethics Statement

The study protocol was approved by the Ethics Committee of the University of Pavia on October 6th, 2009 (Committee report 3/2009). In case of deceased subjects, the consent is not required, as the samples had been taken anyway for clinical/forensic purposes and because it is not possible to contact the next of kin in such circumstances. The reference law is the authorization n9/2016 of the guarantor of privacy, then replaced by REGULATION (EU) 2016/679 OF THE EUROPEAN PARLIAMENT AND OF THE COUNCIL.

## CRediT authorship contribution statement

**Maria Garofalo:** Writing – original draft, Methodology. **Stella Gagliardi:** Writing – original draft, Methodology. **Susanna Zucca:** Software, Writing – original draft. **Cecilia Pandini:** Methodology, Data curation. **Francesca Dragoni:** Methodology, Data curation. **Daisy Sproviero:** Writing – review & editing. **Orietta Pansarasa:** Writing – review & editing. **Tino Emanuele Poloni:** Writing – original draft. **Valentina Medici:** Writing – original draft. **Annalisa Davin:** Writing – review & editing. **Silvia Damiana Visonà:** Data curation. **Matteo Moretti:** Data curation. **Antonio Guaita:** Supervision. **Mauro Ceroni:** Supervision. **Livio Tronconi:** Supervision. **Cristina Cereda:** Supervision, Writing – review & editing.

## Declaration of Competing Interest

The authors declare that they have no known competing financial interests or personal relationships which have or could be perceived to have influenced the work reported in this article.

## Appendix Supplementary materials

Image, application 1

Image, application 2

## References

[1] Gagliardi S., Poloni E.T., Pandini C., Garofalo M., Dragoni F., Medici V., Davin A., Visonà S.D., Moretti M., Sproviero D., Pansarasa O., Guaita A., Ceroni M., Tronconi L., Cereda C. (2021). Detection of SARS-CoV-2 genome and whole transcriptome sequencing in frontal cortex of COVID-19 patients. Brain. Behav. Immun..

[2] Smith T., Heger A., Sudbery I. (2017). UMI-tools: modeling sequencing errors in unique molecular identifiers to improve quantification accuracy. Genome Res..

